# How GWEPs Are Impacting Age Friendly and Dementia-Friendly Communities: Baystate Health

**DOI:** 10.1093/geroni/igaa057.1775

**Published:** 2020-12-16

**Authors:** Maura Brennan

**Affiliations:** Baystate Health, Northampton, Massachusetts, United States

## Abstract

Baystate has collaborated with Community Based Organizations (CBOs) to secure designation for Springfield, MA as both age and dementia friendly. We worked together so our city could be recognized as the first in the nation which was age and dementia friendly and also had an age-friendly health system within it. Baystate joined a Springfield coalition of CBOs; with the assistance of the Massachusetts Healthy Aging Coalition, AARP, State and local Elder Affairs, the Massachusetts Councils on Aging and the Institute for Healthcare Improvement, we secured and celebrated all three recognitions at a public forum in June 2019. The event was attended by the Mayor, Baystate Health and local Elder Affairs leaders, the press and other stakeholders as well as older adults from the community. Along with ongoing efforts to improve transportation and housing, access to age-friendly health care is now also an additional area of focus for the coalition.

